# Metabolic stress reveals widespread accumulation of cap-unmethylated RNAs

**DOI:** 10.64898/2026.02.23.707474

**Published:** 2026-02-24

**Authors:** Zheng Xing, Anna Violet Freitas, Benjamin M. Sutter, Ngoc Khoi Dang, Nicholas T. Ingolia, Benjamin P. Tu

**Affiliations:** 1 Department of Biochemistry, University of Texas Southwestern Medical Center, Dallas, TX, USA; 2 Department of Molecular and Cell Biology, University of California, Berkeley, Berkeley, CA, USA; 3 Lyda Hill Department of Bioinformatics, University of Texas Southwestern Medical Center, Dallas, TX, USA; 4 Center for Computational Biology and California Institute for Quantitative Biosciences, University of California, Berkeley, Berkeley, CA, USA; 5 Howard Hughes Medical Institute, University of Texas Southwestern Medical Center, Dallas, TX, USA

## Abstract

RNA polymerase II transcripts are capped with N^7^-methylguanosine (m^7^G), a conserved modification essential for mRNA function. Although traditionally viewed as constitutive, we developed a mass spectrometry-based method to demonstrate that in both yeast and mammalian cells, a substantial population of mRNAs lack cap methylation in response to SAM-limiting conditions and oxidative stress, which may be frequently encountered across organisms. Through developing two transcriptome-wide approaches, we found that methylation is enriched on specific transcripts and uncovered an unexpected connection between histone H3K36me3 and cap methylation, with both marks preferentially associated with stress-responsive MAPK signaling pathways. Strikingly, cap-unmethylated mRNAs exhibit features of canonical mRNAs—they are polyadenylated, exported to the cytosol, and translated. Enforced cap methylation reduces cell growth under SAM limitation, suggesting that unmethylated mRNAs confer an adaptive advantage during stress. These findings establish mRNA cap methylation as a dynamic, regulated modification and a previously underappreciated layer of gene expression control.

## Introduction

The metabolism of mRNAs represents a key aspect in the regulation of gene expression and cellular functions. A eukaryotic mRNA consists of several components: a 5’ cap, 5’ and 3’ untranslated regions (UTRs), a coding region, and a 3’ poly(A) tail. Decades of research have established that many of these components are subject to regulatory control. For example, the UTRs and poly(A) tail can vary in length in response to stress conditions^1,2^. Poly(A) tails can undergo deadenylation and re-adenylation as part of the post-transcriptional regulation of maternal mRNAs in the oocyte-to-embryo transition^3^. Introns can be alternatively spliced as a means of gene expression regulation^4^. However, fewer mechanisms have been attributed to regulation of the mRNA cap modification itself.

Recent years have seen discoveries of non-canonical cap structures derived from metabolites, such as nicotinamide adenine dinucleotide (NAD^+^) and flavin adenine dinucleotide (FAD)^5^. Metabolite caps are not prevalent compared to canonical cap structures (~1000-fold lower in amount) in human cells^6^. The abundance of metabolite caps correlates with the cellular levels of the corresponding metabolites^5^, suggesting that mRNA cap composition could change according to the metabolic status of the cell, and possibly alter mRNA metabolism. Intriguingly, the most abundant cap structure in the cell, the m^7^GpppN cap, requires one key metabolite, SAM, for its synthesis. The methylation of the mRNA cap has long been recognized as critical for mRNA processing and function, particularly for canonical translation, owing to its recognition by the initiation factor eIF4E^7^. SAM is the methyl currency that links environmental conditions to the growth or survival state of the cell^8^. Genes encoding SAM synthetases have been identified as high copy suppressors for a conditional cap methyltransferase (*ABD1*) mutant in yeast^9^, suggesting that cap methylation by Abd1 may be sensitive to SAM levels.

Since its discovery in the 1970s^10^, the m^7^G cap has been generally considered a constitutive modification. In the last two decades, however, several lines of evidence suggest the existence of cap-unmethylated mRNAs. Overexpression of gene-specific transcription factors c-Myc or E2F1 increases m^7^G-capped RNA levels on several of their target genes^11–13^. Phosphorylation of the mammalian cap methyltransferase (RNMT) was shown to affect the m^7^G-capped levels of three mRNAs tested^14^. However, these studies relied on anti-m^7^G antibodies, which may cross-react with internal m^7^G modifications present in mammalian RNAs^15^. More recently, a mass spectrometry-based method (CAP-MAP) detected unmethylated caps in RNMT or RNMT-activating miniprotein (RAM) knockout cells^16,17^. These studies collectively show that RNMT activity can be regulated. However, the consensus view in mRNA metabolism is that transcripts lacking cap methylation are rare and degraded. In line with this, the DXO family of decapping enzymes and exonucleases are proposed to act as a quality control mechanism to clear mRNAs with GpppN unmethylated caps *in vivo*^18–20^. Here, we investigate the dynamics of mRNA cap methylation and the functional roles of unmethylated GpppN-capped mRNAs. We find that cap-unmethylated transcripts become prevalent under metabolic stress, such as SAM restriction. Our results demonstrate that cap methylation is a regulated process, with a subset of mRNAs governing stress responses displaying preferred methylation, and that unmethylated mRNAs can remain functional and play critical roles in cellular adaptation under such conditions.

## Results

### GpppN-capped mRNAs can accumulate in both yeast and mammalian cells

**Fig. 1.**
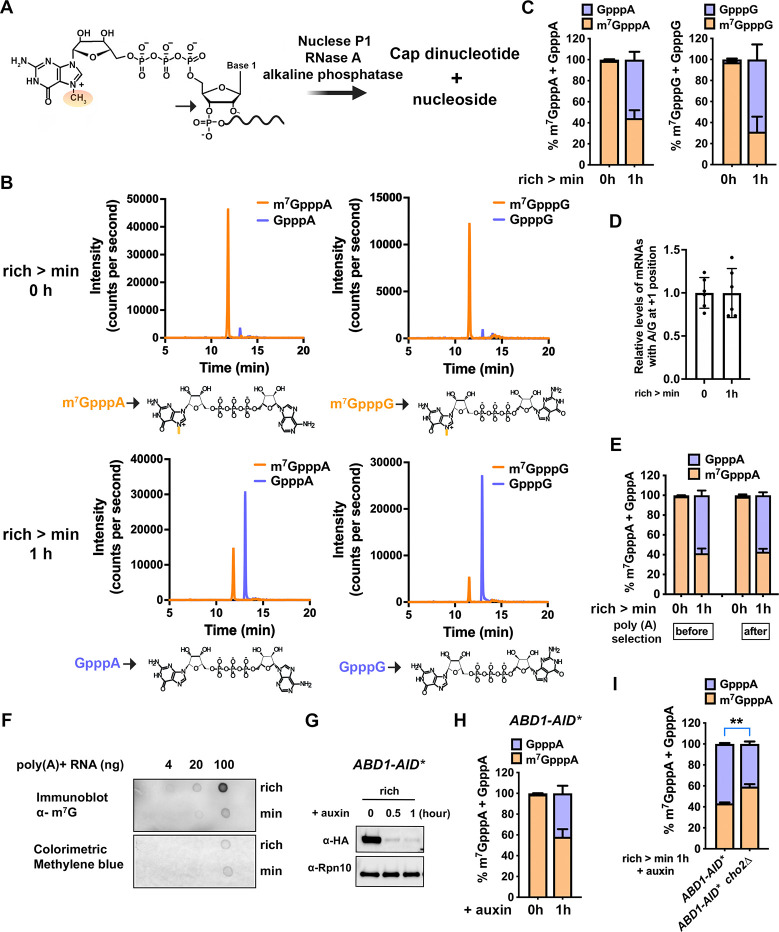
Dynamic accumulation of GpppN-capped mRNAs. **(A)** Schematic of mRNA digestion releasing cap dinucleotides and nucleosides. **(B)** LC-MS/MS chromatograms of cap dinucleotides from mRNAs of cells grown in rich (YPL, top) or minimal (SL, bottom) medium for 1 h. Structures of the species representing major peaks are shown below each panel. **(C)** Quantification of cap dinucleotide structures in rich vs. minimal medium using external standards (Fig. S1A). **(D)** Relative abundance of total mRNAs with A or G at +1 position, measured by LC-MS/MS and normalized to total adenosine. **(E)** GpppA and m^7^GpppA cap percentages before/after oligo dT selection. **(F)** Dot-blot of poly(A)-selected mRNAs of cells grown in rich or minimal medium, probed with m^7^G antibody and methylene blue stain. **(G)** Western blot showing Abd1-AID*-HA depletion over time with auxin (200 μM). **(H)** Percentage of GpppA or m^7^GpppA cap structures before and after Abd1 depletion for 1 h. **(I)** Percentage of GpppA cap structures of ABD1-AID* and ABD1-AID*cho2Δ cells, after depletion of Abd1 for 1 hour. All bar graph data represent ≥ 3 biological replicates and are shown as mean ± standard deviation (SD). *, **, *** indicate p < 0.05, < 0.01, < 0.001, respectively.

We employed a previously established method to induce methionine/SAM restriction in yeast by shifting a prototrophic strain from rich lactate medium (YPL) to synthetic minimal lactate medium (SL)^21^. To quantitate cap methylation of mRNAs, we digested cellular RNAs and quantified cap dinucleotide structures in mRNAs using liquid chromatography–tandem mass spectrometry (LC-MS/MS) (Fig. 1A, B). Using external standards (Fig. S1A), we quantified cap structures with the first transcribed nucleotide (+1) being a purine, as purines—particularly adenine—are more common than pyrimidines at the +1 position^22^. Unexpectedly, GpppN-capped mRNAs accumulated to ~50% after 1 hour of starvation (Fig. 1C), while total mRNA levels remained unchanged (Fig. 1D). As m^7^GpppA and m^7^GpppG caps behave similarly in our assays, we present only m^7^GpppA cap data in subsequent experiments. To confirm that GpppN-capped mRNAs are polyadenylated, we performed oligo(dT) selection and observed no change in the percentage of GpppA-containing transcripts, indicating that these unmethylated mRNAs possess poly(A) tails (Fig. 1E). This accumulation was further validated by dot blot using an m^7^G-specific antibody with poly(A)+ transcripts (Fig. 1F).

The essential protein Abd1 is the sole cap methyltransferase in yeast^23^. Depletion of Abd1 using an auxin-inducible degron (AID*) (Fig. 1G)^24^ led to accumulation of GpppA-capped mRNAs (Fig. 1H). This accumulation was partially rescued by increasing cellular SAM levels via deletion of the phosphatidylethanolamine methyltransferase (PEMT) *CHO2*, which was shown to be a major consumer of SAM for phospholipid methylation (Fig. 1I)^25^. These findings demonstrate that mRNA cap methylation is sensitive to cellular SAM levels and cap methyltransferase abundance. Importantly, methionine/SAM starvation did not alter Abd1 levels (Fig. S1B), confirming that GpppA mRNA accumulation under SAM limitation is not due to changes in Abd1 expression.

We also observed that methionine starvation in mammalian cell cultures leads to the accumulation of cap-unmethylated mRNAs (Fig. S1C, D). Unlike yeast, mammalian mRNAs contain additional cap modifications, including 2′O-methylation at the +1 nucleotide (cap 1) and N^6^-methylation of +1 adenosine, resulting in a spectrum of cap structures—from fully methylated to completely unmethylated GpppN. For subsequent investigations, we decided to focus on the simpler yeast system, which possesses only the m^7^G cap (cap 0) structure^10^.

In principle, GpppN-capped mRNAs could arise during new transcription or as a result of demethylation of an m^7^G-capped mRNA. To this end, we tested whether the GpppN mRNAs we observed were newly synthesized. Upon switching cells from rich to minimal medium, we added [^13^C]-labeled guanine (+3) and readily detected heavy-labeled cap dinucleotides using LC-MS/MS (Fig. S1E), revealing that both m^7^GpppA and GpppA mRNAs can be newly synthesized. Incomplete labeling likely reflects limited incorporation of heavy guanine into guanosine (Fig. S1F). We next added the transcription inhibitor thiolutin when switching cells to minimal medium, which reduced the accumulation of GpppA mRNAs (Fig. S1G), suggesting that they arise from new transcription. Lastly, deletion of individual genes encoding known demethylase enzymes did not affect GpppA mRNA accumulation (Fig. S1H). As m^7^G cap-specific demethylases have not yet been identified in eukaryotes, these data suggest that GpppN mRNAs arise from new transcription rather than demethylation of existing caps.

Rai1 and Dxo1 are yeast decapping and exonuclease enzymes that act on nonconventional mRNA cap structures *in vitro*, including the GpppN cap^19^. They are proposed to serve as quality control factors that clear GpppN-capped mRNAs *in vivo*. To test this, we generated single and double deletion mutants of *RAI1* and *DXO1*. Surprisingly, these deletions did not lead to increased GpppA accumulation (Fig. S1I). To test whether the endogenous levels of Rai1 and Dxo1 are insufficient to promote GpppN turnover, we overexpressed both genes under the constitutive *TEF1* promoter but observed no change in GpppA accumulation (Fig. S1J, K). Thus, clearance of transcripts with unmethylated caps may not represent the primary role of these enzymes in our experimental conditions.

### Cap methylation is enriched for a subset of mRNAs.

**Fig. 2.**
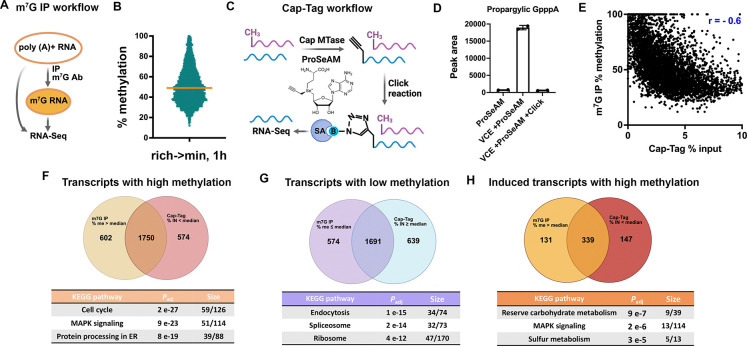
m^7^G cap methylation exhibits transcript-specific distribution. **(A)** Schematic of m^7^G IP workflow. **(B)** Scatter plot showing distribution of mRNA methylation levels after 1 h in minimal medium; yellow line indicates median. **(C)** Schematic of Cap-Tag workflow. **(D)** LC-MS/MS analysis of propargylic GpppA after treatment of poly(A)+ RNA with: ProSeAM only (bar 1); VCE + ProSeAM (bar 2); and VCE + ProSeAM followed by Click biotinylation, which diminishes propargylic GpppA (bar 3); n=2. **(E)** Correlation between % methylation (m^7^G-IP) and % input (Cap-Tag); Pearson r was calculated from 5,155 mRNAs. **(F-H)** Venn diagrams showing overlap of high-/low-methylation transcripts identified by both methods, or highly methylated transcripts that are also induced by 2-fold. High-confidence (overlapping) transcripts were used for KEGG enrichment analysis via YeastEnrichr^47^. Size shows the numbers of genes in the high confidence list/number of genes in the KEGG term.

If cap methylation is a dynamically regulated process, methylation amounts are likely gene-specific rather than uniform. To measure the extent of cap methylation on individual mRNAs, we developed an m^7^G immunoprecipitation (m^7^G-IP) method, utilizing an antibody (K121) that specifically recognizes N^7^-methylated guanosine to enrich for m^7^G-capped mRNAs from the poly(A)+ RNA pool (Fig. 2A). To validate antibody specificity, we demonstrated that K121 binding to mRNA is competed by methylated, but not unmethylated, cap analogs (Fig. S2A). It also exhibits increased mRNA affinity as compared to IgG control (Fig. S2B), and binds selectively to m^7^G-capped *in vitro* transcribed RNA (Fig. S2C). We quantified cap methylation by comparing read counts before (input) and after (IP) enrichment. By arbitrarily setting cap methylation in rich medium to 100%, we were able to estimate the % methylation for each transcript following 1 hour of methionine/SAM starvation. This revealed that m^7^G is not uniformly distributed across mRNAs, but is instead enriched on a specific subset of transcripts. Meanwhile, a large proportion of transcripts exhibit ~40–50% methylation, consistent with the overall bulk cap methylation level (Fig. 2B).

In addition to the 5′ cap, m^7^G can occur internally within mRNAs^15^. While internal m^7^G is absent in *S. cerevisiae*^26^, it has been reported in mammalian cells^15^. To provide an alternative method compatible with mammalian systems and to further validate our m^7^G-IP results, we developed the *in vitro* Cap-Tag assay. Briefly, poly(A)+ RNAs were incubated with a cap methyltransferase and the SAM analog ProSeAM^27^. This incorporates a propargyl group into GpppN-capped mRNAs, while not affecting the m^7^GpppN-capped mRNAs. The propargyl group enables click chemistry-based biotinylation and subsequent enrichment of transcripts that lack cap methylation. Biotinylated mRNAs are captured with streptavidin beads and sequenced (Fig. 2C). LC-MS/MS confirmed propargyl incorporation into GpppA-capped mRNAs, and the click reaction reduced signal intensity, consistent with successful biotin addition (Fig. 2D). *In vitro* transcribed, GpppG-capped RNA and mRNAs from cells grown in rich medium were tested as negative controls and showed minimal enrichment (Fig. S2D, E). Cap-Tag was performed using poly(A)+ RNAs from cells subjected to 1 h of methionine/SAM starvation. Control samples omitting the cap methyltransferase were used to estimate background and subtracted from the final data. We calculated % input to represent GpppN enrichment and compared this to % methylation from m^7^G-IP for each transcript. The two datasets showed strong anticorrelation (Fig. 2E), consistent with the enrichment of methylated mRNAs by m^7^G-IP and unmethylated mRNAs by Cap-Tag.

We defined high- and low-methylation transcripts using the median value from each dataset and identified overlapping genes as high-confidence targets. A substantial overlap was observed between the two datasets (Fig. 2F–H). Pathway enrichment analysis revealed that dynamic processes such as cell cycle and MAPK signaling are enriched in genes with highly methylated mRNAs (Fig. 2F), and terms such as endocytosis, spliceosome, and ribosome, are enriched in genes with lowly methylated mRNAs (Fig. 2G). Although cap methylation levels did not correlate with mRNA abundance or induction (Fig. S2F, G), we sought to exclude the influence of long-lived transcripts synthesized and methylated prior to starvation. To this end, we repeated the pathway enrichment analysis using only genes with induced expression. This analysis revealed enrichment for reserve carbohydrate metabolism, MAPK signaling, and sulfur metabolism (Fig. 2H)—pathways closely associated with nutrient stress responses. Taken together, we have confirmed that cap methylation is selectively enriched in a subset of mRNAs during methionine/SAM starvation.

### Cap methylation exhibits a robust correlation with elevated H3K36me3 levels, and is linked to MAPK signaling

**Fig. 3.**
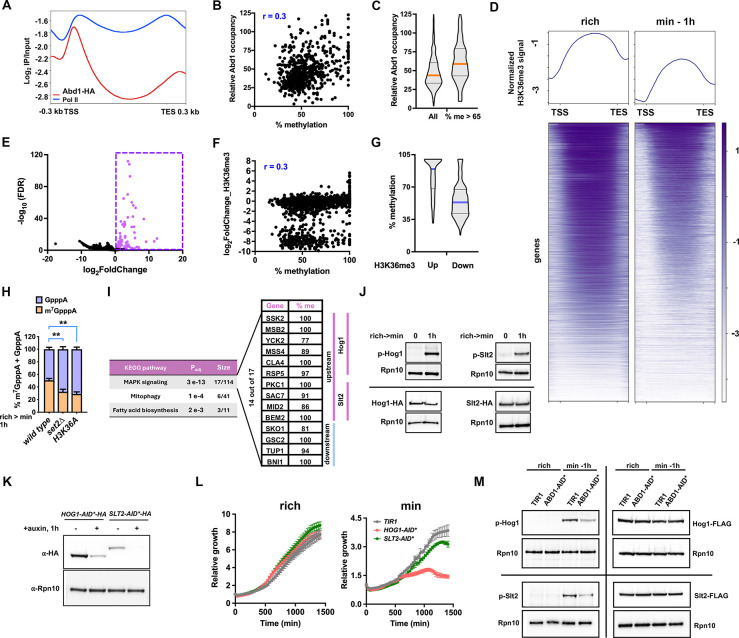
Selective mRNA cap methylation associates with differential Abd1 binding and H3K36me3 levels. **(A)** Metagene profiles showing average Abd1-HA and Rpb1 (Pol II) occupancy in cells grown in minimal medium for 1 h. **(B)** Correlation between relative Abd1 occupancy (normalized to Pol II) and percent cap methylation (Pearson r = 0.3). **(C)** Violin plots comparing relative Abd1 occupancy in all mRNAs versus those with >65% cap methylation (orange line, median). **(D)** H3K36me3 ChIP-seq profiles in rich or minimal medium, normalized to total H3. **(E)** Volcano plot highlighting 227 genes (magenta) with increased H3K36me3 after 1 h in minimal medium (log_2_FC > 0, FDR < 0.05). **(F)** Correlation between log_2_FC in H3K36me3 and percent cap methylation (Pearson r = 0.3). **(G)** Violin plots comparing percent cap methylation in genes with upregulated H3K36me3 versus all genes. **(H)** Percentage of GpppA or m^7^GpppA cap structures in wild type, *set2*Δ, and *H3K36A* mutant after 1 h in minimal medium. mean ± SD, n = 4. ** indicates *p* < 0.01. **(I)** KEGG pathways enriched among genes with increased H3K36me3 upon methionine starvation. Size shows (H3K36me3-upregulated genes)/(total genes in pathway). **(J)** Western blot of Hog1 and Slt2 phosphorylation after 1 h in minimal medium; separate HA blots show total protein levels remain unchanged. **(K)** Western blot showing depletion of Hog1 and Slt2 using the AID* system. **(L)** Growth curves of *TIR1* and Hog1 or Slt2-depleted cells in rich or minimal media; mean ± SD, n = 6. **(M)** Western blot showing Hog1 and Slt2 phosphorylation after 1 h in minimal medium in both *TIR1* and Abd1-depleted strains; FLAG-blots show total Hog1 and Slt2 protein levels remain unchanged after the media switch.

How is differential methylation of specific mRNAs achieved? Abd1 is thought to methylate the mRNA cap co-transcriptionally by binding to the C-terminal domain of RNA polymerase II (Pol II)^28^. We performed chromatin immunoprecipitation followed by sequencing (ChIP-seq) against Abd1-HA and Rpb1, a subunit of the RNA Pol II holoenzyme. Consistent with prior findings, Abd1 signal peaks at the 5′ ends of genes (Fig. 3A) and shows a strong genome-wide correlation with Pol II occupancy (Fig. S2H). To assess Abd1 binding without influences by transcriptional activities, we quantified relative Abd1 occupancy as the ratio of Abd1/Pol II signal. This metric shows a weak to moderate correlation with the percentage of cap methylation (Fig. 3B), with highly-methylated mRNAs exhibiting higher relative Abd1 occupancy (Fig. 3C). These data suggest that cap methylation may be, in part, linked to differential Abd1 recruitment.

We previously demonstrated that bulk histone methylation is responsive to cellular SAM levels, with global histone methylation—and particularly H3K36me3—reduced upon SAM starvation^29^. To investigate whether mRNA cap methylation correlates with histone methylation, we performed ChIP-seq for H3K4me3, H3K36me3, and H3K79me3. As expected, these marks show canonical distributions—H3K4me3 enriched at transcription start sites (TSSs) and H3K36me3/H3K79me3 across gene bodies. Consistent with earlier results, we observed the most pronounced global reduction in H3K36me3 upon starvation (Fig. 3D and Fig. S2I, J). Surprisingly, a subset of genes displayed a marked increase in H3K36me3 (Fig. 3E). Changes in H3K36me3 show a weak to moderate correlation with mRNA cap methylation (Fig. 3F). Notably, elevated H3K36me3 strongly predicts high levels of cap methylation (Fig. 3G). Consistent with this observation, strains lacking the H3K36 methyltransferase *SET2*^30^ or harboring the non-methylatable *H3K36A* mutation displayed reduced m^7^GpppA percentages (Fig. 3H), suggesting that H3K36 tri-methylation enhances cap methylation for a subset of transcripts.

Pathway analysis of these genes identified MAPK signaling as the top enriched pathway. Among 17 MAPK pathway genes with elevated H3K36me3, 14 also exhibited high cap methylation (Fig. 3I). Examination of these highly cap-methylated transcripts revealed enrichment for regulators in the Hog1 and Slt2 MAPK branches, with 10 genes positioned upstream of Hog1 or Slt2. In line with this, Hog1 and Slt2 were phosphorylated upon switching to minimal medium (Fig. 3J), and depletion of these kinases—especially Hog1—compromised growth in minimal but not rich medium (Fig. 3K, L). Moreover, disruption of cap methylation by depleting Abd1 reduced Hog1 and Slt2 phosphorylation after the media switch without affecting their total protein levels (Fig. 3M), supporting a role for prioritized cap methylation in promoting MAPK activation. Together, these findings indicate that both H3K36me3 and mRNA cap methylation respond similarly to SAM starvation: they are globally downregulated but selectively maintained or elevated at a shared subset of genes, such as genes involved in MAPK signaling—a pathway critical for protecting cells from damaging stress.

### GpppN mRNAs can be translated and are critical during SAM starvation and oxidative stress

**Fig. 4.**
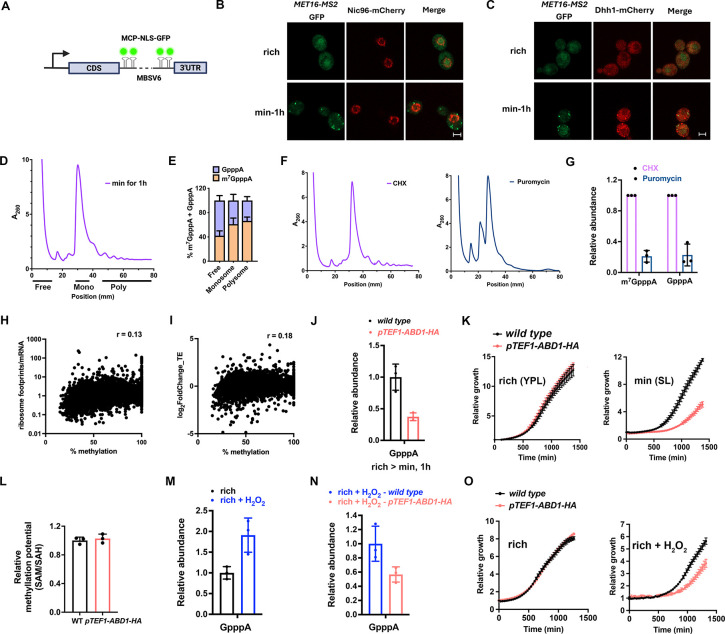
GpppN-capped mRNAs are functional. **(A)** Schematic of MS2-based visualization of an mRNA of interest. **(B)** Live confocal images of MET16 mRNA (MCP-NLS-eGFP, green) and nuclear pores (Nic96-mCherry, red); scale bar, 2 μm. **(C)** Live confocal images of MET16 mRNA (green) and P-body marker Dhh1-mCherry (red); scale bar, 2 μm. **(D)** Polysome profiling of cells grown in minimal medium for 1 h. **(E)** LC-MS/MS quantification of GpppA and m^7^GpppA in pooled fractions from (D); mean ± SD, n = 3. **(F)** Polysome profiling of cells in minimal medium (1 h) with CHX (0.1 mg/mL) or puromycin (2 mM) treatment. **(G)** Relative abundance of m^7^GpppA and GpppA after CHX (set to 1) or puromycin treatment, normalized to adenosine; mean ± SD, n = 3. **(H)** Correlation of ribosome footprint/mRNA reads and methylation levels of each mRNA in cells grown in minimal medium for 1 h. Pearson r = 0.13. **(I)** Correlation of log_2_FC of TE (min 1h/rich) and % methylation levels of each mRNA in cells grown in minimal medium for 1 h. Pearson r = 0.18. **(J)** LC-MS/MS analysis of relative GpppA levels in minimal medium (1 h) for wild type and *ABD1* overexpression strains; mean ± SD, n = 3. **(K)** Growth curves of wild type and *ABD1* overexpression strains; mean ± SD, n = 4. **(L)** Relative methylation potential (SAM/SAH ratio) in wild-type and *ABD1* overexpression strains. **(M)** LC-MS/MS analysis of relative GpppA levels in rich medium ± 1 mM H_2_O_2_ (1 h); mean ± SD, n = 3. **(N)** LC-MS/MS analysis of relative GpppA levels of wild type and *ABD1* overexpressing cells grown in rich medium + 1 mM H_2_O_2_ for 1 h; mean ± SD, n = 3. **(O)** Growth curves of wild type and *ABD1* overexpressing cells, mean ± SD, n = 3.

In addition to investigating the regulation of selective cap methylation, we also characterized GpppN-capped mRNAs, which represent a previously underappreciated class of mRNAs in the cell. The observation that mRNAs are not uniformly methylated and that cap-unmethylated mRNAs are not rapidly degraded suggest that they may be functional. To investigate their fate, we first examined the localization of cap-unmethylated mRNAs. We inserted MS2 binding sites (MBSV6^31^) into the 3’UTR of the *MET16* mRNA, a lowly methylated transcript during SAM starvation (% methylation ~ 26%, Supplemental Data 1) (Fig. 4A). This enabled visualization of *MET16* mRNA via expression of MS2 coat protein (MCP)-GFP. Interestingly, *MET16* mRNA appears as cytoplasmic foci in live cells (Fig. 4B), a finding corroborated by FISH in fixed cells (Fig. S3A). As stress granules and processing-bodies (P-bodies) are common Ribonucleoprotein (RNP)-containing foci, we assessed their formation under methionine starvation. Only P-bodies formed under these conditions (Fig. S3B, C), yet *MET16* mRNA foci did not colocalize with P-bodies (Fig. 4C). Similarly, two other lowly methylated mRNAs, *BET1* and *RPN12* (~33% and 29% methylation, respectively), also formed distinct cytoplasmic foci (Fig. S3D, E). These results suggest that cap-unmethylated mRNAs are exported to the cytoplasm and do not localize to known RNP granules.

We next asked if cap-unmethylated mRNAs have the potential to be translated. We performed polysome profiling on cells grown in minimal medium for 1 h, extracted RNA from fractions representing the free mRNA, monosome, and polysome populations (Fig. 4D), and analyzed cap structures by LC-MS/MS. Surprisingly, we observed that GpppA mRNAs are present in both the monosome and polysome fractions (Fig. 4E). Northern blot analysis confirmed the presence of the lowly methylated *MET16* mRNA in these fractions (Fig. S3F). Furthermore, we treated the cell lysate with puromycin to dissociate mRNAs from the polysome fractions (Fig. 4F)^32^. This treatment reduced the polysome association of both m^7^GpppA and GpppA mRNAs (Fig. 4G), validating that GpppA mRNAs associate with active polysomes and suggesting that they are translated.

To investigate the global relationship between cap methylation and translation efficiency (TE), we performed ribosome profiling on cells collected before and after methionine starvation. TE was calculated as the ratio of ribosome footprint reads to mRNA reads. This analysis revealed a weak positive correlation between TE and mRNA cap methylation levels (correlation coefficient r ~ 0.13; Fig. 4H), as well as between changes in TE after starvation and cap methylation levels (r ~ 0.18; Fig. 4I). In addition, changes in protein abundance showed essentially no correlation with cap-methylation levels (r ~ 0.03, Fig. S3G). These results support the notion that cap-unmethylated mRNAs are translated, albeit likely at lower rates than fully methylated transcripts. To directly test the effect of cap methylation on translation, we *in vitro* transcribed and polyadenylated *MET16* RNA containing its native UTRs and coding region, along with a FLAG tag sequence. Transcripts were capped with either m^7^GpppG or GpppG (Fig. S3H) and subjected to *in vitro* translation using yeast extracts. Western blot analysis showed that GpppG-capped *MET16* RNA was translated, but with lower efficiency compared to m^7^GpppG-capped RNA (Fig. S3I).

Switching yeast cells from rich to minimal medium induces acute methionine/SAM starvation. However, yeast cells can recover from this stress by reprogramming their transcription and metabolism, allowing synthesis of methionine/SAM *de novo* using inorganic sulfur^8^. This transition represents a critical period when the cells undergo active adaptation. To test whether the accumulation of GpppN-capped mRNAs might be beneficial during this phase, we overexpressed *ABD1* under the constitutive *TEF1* promoter. We confirmed that the GpppA cap levels are reduced upon Abd1 overexpression (Fig. 4J). Interestingly, this impaired the growth of cells in minimal medium but not in methionine/SAM-replete rich medium (Fig. 4K). This growth defect is not due to change in cellular methylation potential, as SAM/SAH levels remained unchanged upon Abd1 overexpression (Fig. 4L). We also showed that expression of the SARS-CoV2 viral cap methyltransferase NSP14 reduces growth of yeast cells in minimal medium but not rich medium (Fig. S3J–L). NSP14 expression decreases GpppA-mRNA levels, consistent with its ability to methylate yeast host mRNA caps (Fig. S3M)^33^. This raises the possibility that viral infection could alter cellular mRNA cap methylation status, representing a previously unrecognized consequence of viral infection.

To explore conditions that may promote the accumulation of cap-unmethylated mRNAs beyond nutrient deprivation, we treated cells with H_2_O_2_, an oxidant that may perturb SAM homeostasis^8^, which increased GpppA-mRNA levels (Fig. 4M). Consistent with our findings above, overexpression of Abd1 during H_2_O_2_ exposure reduced GpppA accumulation (Fig. 4N), which was accompanied by reduced cell growth (Fig. 4O). Taken together, these results suggest that the accumulation of GpppN-capped mRNAs confer a growth advantage under stress conditions.

## Discussion

We demonstrate for the first time that cap-unmethylated mRNAs can be prevalent in cells, especially under conditions where SAM is limiting, opening a new avenue for exploring their biological functions and cap methylation dynamics. This finding also raises several immediate questions, which we discuss below.

Why does increased H3K36me3 predict higher cap methylation? We observed an intriguing clustering of elevated H3K36me3 signals during methionine starvation, with certain chromosomal regions forming distinct clusters of H3K36me3 enrichment (Fig. S4A). One possible explanation is that these genomic regions are also spatially clustered within the nucleus and reside in compartments enriched in local SAM levels. Consequently, these regions may experience elevated levels of both histone and cap methylation. In addition, the reduced m^7^GpppA–mRNA levels detected in the *H3K36A* mutant suggest that the H3K36 methylation state itself may contribute to enhanced cap methylation.

The observation that mRNA expression and induction levels do not correlate with cap methylation levels suggests that transcription and cap methylation are not necessarily coupled processes. This prompts a key question: why do cells transcribe certain mRNAs without methylating their caps? Here, we provide the first evidence that cap-unmethylated mRNAs share key features with methylated ones—they are polyadenylated, exported, and translated. As for pre-mRNA splicing, about 280 genes in *S. cerevisiae* contain introns^34^, limiting the scope of pre-mRNA splicing regulation and alternative splicing. Nevertheless, we quantified ratios of intronic to exonic reads in input, m^7^G-IP, and Cap-Tag samples (Fig. S4B, C). Cap-Tag libraries showed an enrichment of intronic reads, whereas m^7^G-IP libraries contained fewer intronic reads. This pattern is exemplified by normalized RNA-seq coverage tracks of the intron-containing gene *YRA1* (Fig. S4D, E). While alternative explanations cannot be ruled out, the data indicate that cap-unmethylated mRNAs are spliced less extensively than their cap-methylated counterparts. Most importantly, cap-unmethylated transcripts appear critical for cell growth under stress. Based on our findings, we propose several hypotheses regarding the underlying mechanisms: cap methylation status could represent an additional strategy to modulate translation efficiency, as cap-unmethylated mRNAs typically exhibit reduced translation efficiency compared to their methylated counterparts^35^. Cytosolic recapping and cap methylation have been reported in mammalian cells^36^. Consistent with this possibility, the lack of a growth defect upon adding a nuclear export signal to Abd1 (Fig. S4F) suggests that cytosolic cap methylation may also occur in yeast. This could allow existing unmethylated transcripts to be rapidly methylated and translated faster in response to environmental cues. Interestingly, ribosome-related transcripts are among the least methylated (Fig. 2G), and their translation is highly correlated with cell growth. These mRNAs may represent a class of transcripts that are “pre-synthesized and stored,” allowing for rapid expression once nutrient conditions improve, potentially conferring a growth advantage to the cell.

Intriguingly, we also identified a subset of mRNAs that retain robust TE following a shift to minimal medium (Fig. S4G, H). We focused on mRNAs with both increased expression and strong TE, reasoning that these mRNAs are more likely to represent robust cases. These findings suggest the possibility of an alternative translation initiation mechanism that may facilitate translation of unique cap-unmethylated mRNAs, warranting further investigation.

Overall, this study represents an initial effort to investigate the dynamic regulation of mRNA cap methylation, and we report the stable existence and potential functionality of cap-unmethylated mRNAs. Our collective observations suggest that both preferentially cap-methylated mRNAs and cap-unmethylated mRNAs are important for the cell to adapt to stress. What might be the key differences between the methylated vs. unmethylated mRNAs? One possibility is that highly methylated transcripts belong to the group of genes that necessitate rapid translation. They could encode proteins that prevent damage to the cell, or perhaps rate-limiting enzymes of a biosynthetic pathway. Cells can then ensure all processes important for survival under stress are prioritized, providing an additional layer of post-transcriptional regulation. For cap-unmethylated mRNAs, enforced cap methylation during metabolic stresses is clearly disadvantageous to cells. We focused on characterizing their fundamental properties by analyzing the bulk population of cap-unmethylated transcripts in this study. However, we anticipate that these mRNAs may serve diverse functional roles under different contexts. To this end, we developed transcriptome-wide methods to assess cap methylation status at the individual mRNA level. These tools will enable the identification of specific transcripts that undergo dynamic cap methylation across a variety of conditions beyond nutrient stress, including developmental stages, disease states, and other physiological or environmental challenges.

## Methods

### Yeast growth.

Yeast cultures were grown in 30°C with 300 rpm shaking. Cultures were typically inoculated the day before, diluted to OD_600_ ~ 0.2 the next morning and allowed to grow to log phase (OD_600_ ~ 0.8) for various experiments. YPL is a rich medium containing 10 g yeast extract (BioBasic, G0961) and 20 g peptone (Bacto, 211677) in 1 L, supplemented with 2% lactate. SL is a minimal medium containing 6.7 g yeast nitrogen base (BD, 291920) in 1 L, with 2% lactate. For media switch, cells were grown in YPL, part of the culture was harvested by centrifugation and flash frozen in liquid nitrogen when applicable, another part was washed once with SL, and resuspended in the same volume of SL. Heavy guanine (Cambridge Isotope Laboratories, CNLM-3990–25), thiolutin (Cayman, 11350), and H_2_O_2_ (Sigma, 216763) were also used when indicated. For serial dilution growth assays, yeast cells were washed with sterile water and spotted on indicated agar plates in 6-fold dilutions, starting with OD_600_ = 0.1. For growth curve analysis, yeast cells were washed with desired growth media and then seeded 100 μL/well in flat bottom 96-well sterile plates (CELLTREAT, 229596). OD_600_ was measured using a Tecan Spark plate reader at 30 min intervals with continuous shaking and magnetic lid lifter for better measurement and aeration.

### Cap LC-MS/MS.

Total RNA was extracted from yeast using the hot acid phenol method. When applicable, poly (A)+ RNA was purified from total RNA using Dynabeads mRNA purification kit (Invitrogen, 61006). 50 μg of total RNA or 1 μg of poly (A)+ RNA was digested in 110 μL reaction containing 7 μL acidic buffer (0.1 M NaOAc pH 6.8, 20 mM ZnCl_2_), 1 μL RNase A (5 μg/μL, Biosearch, MRNA092) and 1 μL Nuclease P1 (1.5 U/μL, Sigma, N8630). After incubation at 37°C for 4 h, 7 μL basic buffer (0.3 M NaOAc pH 7.8) and 1 μL calf intestinal phosphatase (NEB, M0525) was added and the reaction was allowed to continue at room temperature overnight. The reaction was filtered using Amicon ultra 0.5 ml filtration units (10K MWCO). The flow-through, containing the digested nucleosides and cap structures, was collected, and 50 μL was injected into a Synergi 4 μm Fusion-RP 80A 150 X 2 mm column (Phenomenex, 00F-4424-B0) using a Shimadzu high performance liquid chromatography (HPLC). HPLC was operated at 0.5 mL/min with 10 mM tributylamine pH 5 (buffer A) and 100% methanol (buffer B). Gradient elution is performed as follows: 0.01 min, 0% B, 11 min, 50% B, 13 min, 100% B, 21 min, 100% B, 22 min, 0% B, 30 min, 0% B. Analytes were detected using Sciex 5500 QTRAP mass spectrometer with negative mode and multiple reaction monitoring (MRM) using transitions listed below. Analytes were quantified using the Sciex OS software.

### Yeast metabolites analysis.

Metabolites were extracted and dried as described ^40^. Dried metabolites were resuspended in buffer A (5 mM NH_4_OAc pH 5.5 in water) and filtered with a 0.2 μm PVDF syringe filter before injecting into a Synergi 4 μm Fusion-RP 80A 150 X 2 mm column. HPLC was operated at 0.5 mL/min with buffer A and buffer B (5 mM NH_4_OAc in methanol). The following gradient elution was performed: 0.01 min, 0% B, 5 min, 0% B, 6 min, 1% B, 7 min, 3% B, 8 min, 5% B, 14 min, 25% B, 16 min, 50 % B, 18 min, 100% B, 22 min, 100% B, 23 min, 0% B, 28 min, 0% B. Metabolites were detected with 5500 QTrap with positive mode and MRM using transitions listed below, and quantified with Sciex OS.

### Mass spectrometry transitions for cap and metabolites analysis

**Table:** 

Analyte	Mode	Q1	Q3
m^7^GpppA	-	785	408
GpppA	-	771	408
m^7^GpppG	-	801	424
GpppG	-	787	424
m^7^Gpppm^6^Am	-	813	436
m^7^GpppAm	-	799	422
GpppAm^6^Am	-	799	436
GpppAm	-	785	422
m^7^GpppGm	-	815	438
GpppGm	-	801	438
adenosine	-	266	134
guanosine	-	282	150
C13 m^7^GpppA	-	788	620
C13 m^7^GpppA	-	788	408
C13 GpppA	-	774	408
C13 GpppA	-	774	427
C13 guanosine	-	285	153
Propargyl-GpppA	-	809	408
SAM	+	399	250
SAH	+	385	136

### Yeast whole cell lysate preparation and Western blot.

Yeast cells were harvested by centrifugation and resuspended in 20% trichloroacetic acid (TCA) and incubated on ice for 15 min and washed once in cold acetone. TCA-treated cell pellet was lysed by bead-beating (3X 30s) in lysis buffer (50 mM Tris-HCl pH 7.5, 6 M urea, 1% SDS, 5 mM EDTA, 1 mM DTT, 1 mM PMSF, 10 μM leupeptin, 5 μM pepstatin A, and 1X protease inhibitor cocktail (Roche, 11697498001)). After bead-beating, lysate was incubated in 75°C for 5 min, centrifuged at 14000 ×g for 3 min. Supernatant was collected, normalized by total protein concentration using the BCA assay, and used for Western blot analysis. Western blot was done with standard procedures.

### Dot-blot analysis.

Total poly (A)+ RNAs were isolated as described above. mRNAs were diluted to desired concentrations, heated at 70°C for 2 min, snap-cooled on ice, and 2 μL of mRNA solutions were dropped directly onto Hybond-N+ membrane (Cytiva, RPN303B) and allowed to dry briefly. The membrane was crosslinked in a Stratalinker 200 UV crosslinker twice using the auto-crosslink mode (1200 microjoules [x100]). For blotting, the membrane was washed in wash buffer (1x PBS, 0.02% Tween-20) for 5 min at room temperature, incubated with blotting buffer (1x PBS, 0.02% Tween-20, 2% BSA) for 1 h at room temperature, incubated with anti-m^7^G clone K121 antibody (1:35) overnight at 4°C, washed 3X for 5 min each in wash buffer at room temperature, incubated with goat anti-mouse IgG-HRP (1: 10000) for 1 h at room temperature, and washed 4X for 10 min each with wash buffer before imaging using standard Western protocols. To visualize total mRNA on the membrane, a methylene blue solution (0.2 % methylene blue, 0.4 M NaOAc, 0.4 M acetic acid) was incubated with the membrane for 30 min at room temperature and then washed with MilliQ water until desired signal/background reached.

### m^7^G immunoprecipitation.

Total yeast RNA was extracted, treated with Turbo DNase (Ambion, AM2238) and extracted with acid phenol again. Poly(A)+ RNA was enriched as described above. 50 μl of Dynabeads protein G (ThermoFisher, 10003D) was washed 2X with RNA immunoprecipitation (RIP) buffer (25 mM Tris-HCl pH 7.5, 200 mM NaCl, 5 mM EDTA, 0.1% Tween-20, 0.5 mM DTT). To pre-bind antibody, washed beads were mixed with 10 μg m^7^G K121 antibody (Sigma, NA02) or 10 μg mouse IgG in 200 μL of RIP buffer. The mixture was incubated at 4°C for 2 h with rotation, and washed 2X with 500 μL RIP buffer. 300 ng poly(A)+ RNA was added to the beads in 200 μL RIP buffer with 0.1U/μL SUPERase·In (ThermoFisher, AM2696), and incubated at 4°C overnight with rotation. The next day, the beads were washed 3X with 1 mL RIP buffer. Finally, the washed immunoprecipitates were subjected to acid phenol extraction, and precipitated with ethanol and glycogen. The RNA pellet was resuspended in 20 μL water and two biological replicates were subjected to strand-specific RNA-sequencing with Novogene. Genes with fewer than 10 reads in any sample were excluded from further analysis. Data were normalized to the total counts of ERCC RNA spike-ins (ThermoFisher, 4456740). Percent methylation of RNAs from rich medium samples was arbitrarily set to 100% (i.e., (IP^rich^/input^rich^)*X=% methylation^rich^=100%), and % methylation of RNAs from minimal medium samples was calculated using the same scaling factor: % methylation^min^=(IP^min^/input^min^)*X. Because the % methylation in rich medium was arbitrarily set, this calculation yielded 229 transcripts in minimal medium with values exceeding 100%. For these transcripts, the % methylation was capped at 100%.

### *In-vitro* Cap-Tag.

Poly(A)+ RNA was extracted from yeast cells as described above. RNA was DNase I-treated and purified with RNA Clean & Concentrator (Zymo Research, R1017). 500 ng poly(A)+ RNA was labeled by incubating at 37°C for 35 min with 0.05 mM ProSeAM (WuXi AppTec) and 1 μL Vaccinia capping enzyme (NEB, M2080S) (omitted in the background control group) in a 50 μl reaction volume with buffer containing 50 mM Tris-HCl pH 7.5, 5 mM KCl, and 1 mM DTT. Labeled RNA was cleaned with the Zymo RNA Clean & Concentrator kit and eluted in 10 μL water. Click-chemistry reagents were obtained from Jena Bioscience (CLK-071) and reaction was performed per manufacturer instructions. Briefly, 2 μL CuSO4 (100mM) and 20 μL BTTAA (50 mM) were mixed and incubated at room temperature for 5 min. 10 μL labeled RNA, 57.5 μL 100 mM Na-Phosphate buffer pH 7, and 0.5 μL 1 mM Picolyl-Azide-PEG4-Biotin were mixed, and then added to the first part of reaction. 100 mM NaAscorbate was added to start the reaction, and then incubated at 37°C for 35 min. Samples were cleaned with the RNA Clean & Concentrator kit and eluted in 17 μL water. 1 μL of RNA was saved at this step as input.

For each pull-down, 10 μL Dynabeads M-280 Streptavidin (Invitrogen,11205D) was washed 3X with bind buffer (Tris-HCl 50 mM pH 7.5, NaCl 1 M, EDTA 5 mM, Triton X-100 0.2%), blocked for 2 h with blocking buffer (10 mM Tris-HCl pH 7.5, 0.2 mg/mL BSA, 0.2 mg/mL yeast tRNA, glycogen 0.01 mg/mL) at room temperature, and washed 3X again with bind buffer. 15 μL sample from the Click reaction was incubated with the beads in 100 μL bind buffer for 45 min at room temperature. The beads were washed 3X with bind buffer at room temperature, 1X with bind buffer with 4 M NaCl at 42°C for 3min, and 1X with 1 X PBS. The washed beads were then eluted with 50 μL elution buffer (95% formamide, 10 mM EDTA) a 65°C for 5 min. The beads together with the eluate were purified with TRIzol according to manufacturer instructions and two biological replicates were subjected to RNA sequencing with Novogene. After read counting, genes with fewer than 10 reads in any sample were excluded from further analysis. Percent input was calculated using the formula: % input = (count_pulldown / count_input / 22.5) × 100. CleanCap eGFP mRNA (TriLink, L-7601) was spiked into each sample prior to Cap-Tag to estimate background levels, and RNA-seq data from ΔMTase samples were normalized accordingly.

### RNA-sequencing data analysis.

Data analysis was done by Novogene. Briefly, raw data in fastq format was processed and clean reads was obtained by removing reads containing adapter, reads containing poly-N and low-quality reads. Reference genome *Saccharomyces cerevisiae* (R64–1-1) and gene model annotation files were downloaded from ensembl. Index of the reference genome was built, and paired-end clean reads were aligned to the reference genome using Hisat2 v2.0.5. FeatureCounts v1.5.0-p3 was used to count the reads numbers mapped to each gene.

### ChIP-seq and data analysis.

100 OD_600_ units of cells were crosslinked in 1% formaldehyde for 15 min and quenched with 125 mM glycine for 10 min at room temperature. Cell pellets were washed twice with buffer containing 100 mM NaCl, 10 mM Tris-HCl (pH 8.0), 1 mM PMSF, and 1 mM benzamidine-HCl, then snap-frozen in liquid nitrogen. Frozen pellets were resuspended in 0.45 ml ChIP lysis buffer (50 mM HEPES-KOH pH 7.5, 500 mM NaCl, 1 mM EDTA, 1% Triton X-100, 0.1% sodium deoxycholate, 0.1% SDS, 1 mM PMSF, 10 μM leupeptin, 5 μM pepstatin A, and protease inhibitor cocktail [Roche]) and lysed by bead beating. For histone modifications, lysates were sonicated for 16 cycles (30 s on, 1 min off, high output) using a Bioruptor (Diagenode). For Abd1-HA and Rpb1 ChIP samples, sonication was performed with a Pixul system (Active Motif) at 50 pulses, 1 kHz pulse-rate frequency, and 20 Hz burst rate for 40 min. Sonicated lysates were clarified by two sequential centrifugations at 15,000 × g for 10 min each. For Abd1-HA and Rpb1 ChIP, 240 μL of lysate was incubated overnight with 10 μg anti-HA (Cell signaling, 3724S) or 1.5 μg anti-Rpb1 antibody (Biolegend, 664906), respectively. For histone ChIP, 50 μL of lysate was incubated overnight with 2 μg of antibody (abcam, ab8580, ab9050, and ab2621, 05–928). Immunoprecipitated complexes were captured with 25 μL magnetic beads for 1.5 h and washed twice with ChIP lysis buffer, once with 1 mL deoxycholate buffer (10 mM Tris-HCl, 0.25 M LiCl, 0.5% deoxycholate, 1 mM EDTA), and once with 1 mL TE buffer (10 mM Tris-HCl pH 8.0, 1 mM EDTA). Chromatin was eluted with TES buffer (10 mM Tris-HCl pH 8.0, 1 mM EDTA, 1% SDS), and crosslinks were reversed at 65 °C overnight. Eluates were treated with proteinase K (1.25 mg/mL) and RNase A (0.4 mg/mL) for 2 h at 37 °C, and DNA was purified using a QIAGEN PCR purification kit. Two biological replicates for each experiment were sent to Novogene for library construction and sequencing.

ChIP-seq analyses were performed on the BioHPC cluster at UT Southwestern Medical Center. Raw sequencing data were assessed for quality using FastQC (v0.11.9), and adapters and low-quality reads were trimmed with Trim Galore! (v0.6.7). Reads from the Abd1-HA and Rpb1 datasets were aligned to a composite *S. cerevisiae* (sacCer3) and *D. melanogaster* (dm6) reference genome using BWA-MEM2 to enable spike-in normalization. Reads from histone methylation (H3K4me3, H3K36me3, H3K79me3) and total H3 datasets were aligned to the sacCer3. PCR duplicates were removed using the MarkDuplicates tool from Picard (v2.26.10) prior to downstream analyses. Significantly enriched regions were identified with MACS3 (v3.0.0). Peaks for Abd1-HA and Pol II (Rpb1) were called against their respective input DNA controls, whereas histone modification peaks were called against the total H3 control to account for nucleosome occupancy. Signal tracks for visualization were generated using deepTools (v3.5.1). Abd1-HA binding relative to Pol II was quantified as the total Abd1-HA signal across its called peaks divided by the normalized Pol II signal across the gene body. Differential histone modification analysis was performed using DiffBind with a consensus peak set derived from MACS3 outputs. Read counts within consensus peaks were quantified per replicate, and differential enrichment was assessed with the integrated DESeq2 method. Sites with a false discovery rate (FDR) < 0.05 were considered differentially bound.

### Microscopy.

Images were acquired using a Zeiss Axio Observer.Z1 inverted confocal fluorescence microscope with a Plan-Apochromat 100×/1.40 oil DIC M27 objective with immersion oil. For live-cell imaging, yeast cells were grown in appropriate media and imaged immediately at room temperature. Cells grown in YPL were washed in synthetic complete medium supplemented with lactate (SCL) prior to imaging, while cells grown in SL were imaged directly. Imaging was performed with mCherry channel (excitation 587 nm, emission 610 nm; 543–658 nm detection), and eGFP channel (excitation 488 nm, emission 509 nm; 481–543 nm detection) and DAPI channel. For RNA FISH, CAL Fluor Red 610 Dye was detected using the mCherry channel. Images were acquired with confocal pinhole set to 93 μM. Data were acquired and exported using ZEN Blue software. The same brightness and contrast were applied across all images.

### Polysome profiling.

Polysome profiling was performed as described^41^. Briefly, cells were grown at desired conditions and cycloheximide (CHX) was added to 0.1 mg/mL at the time of harvest. Cells were rapidly chilled and incubated on ice for 5 min before centrifugation at 4000 ×g for 2 min. Cell pellet can be frozen in liquid nitrogen and stored in −80°C. Cell pellet was washed 2X with polysome extraction buffer (PEB, 10 mM Tris-HCl pH 7.5, 140 mM KCl, 5 mM MgCl_2_, 1% Triton X-100, 0.5 mM DTT, and 0.1 mg/ml CHX). Washed cells were lysed in PEB by bead beating method (3X, 30s per round with 2 min on ice in between). Lysate was centrifuged at 8000 ×g for 5 min in 4°C and supernatant was collected. A 10%–50% (w/v) sucrose gradient was prepared using BIOCOMP Gradient Station. Cleared lysates (normalized using A260 among samples) were loaded on top of the sucrose gradient and centrifuged in a 12ml centrifuged tube (Seton, 7030) at 41,000×g for 2 h at 4°C. Polysome fractions (0.7 mL each) were collected and A260 was measured using BIOCOMP Gradient Station. For RNA extraction, individual or pooled fractions were precipitated with ethanol and then briefly dissolved in 50 μL water with 100 U/μL rRNasin^®^ (Promega, N2511), followed by RNA isolation with TRIzol reagent per manufacturer instructions.

### Northern blot following polysome profiling.

Northern blotting was performed as previously described^42^. RNA from each fraction was resuspended in equal volume and denatured in formaldehyde loading solution (500 μL formamide, 176 μL 37% formaldehyde, and 50 μL 20× MOPS) at 65–70 °C for 10 min and chilled on ice for 5 min. RNA samples were resolved on a 1.2% denaturing formaldehyde-agarose gel (1.8 g agarose, 7.5 ml 20× MOPS, and 8.04 mL 37% formaldehyde in 150 mL total volume) ran at 4 V/cm for 3 h. After electrophoresis, the gel was soaked in 50 mM NaOH for 20 min and rinsed thoroughly with MilliQ water. Residual formaldehyde was removed by three sequential 5 min rinses in 2× SSC (30 mM sodium citrate pH 7.0 and 300 mM NaCl). RNA was transferred overnight to a Hybond-N+ nylon membrane (Cytiva, RPN303B) via capillary blotting in 10×SSC. After transfer, RNA was UV cross-linked to the membrane (0.12 J, 2X) using a UV crosslinker. Membranes were stained with 0.02% methylene blue in 0.3 M sodium acetate (pH 5.0) to visualize rRNA. For hybridization, membranes were pre-incubated at 65 °C for 1 h in hybridization buffer (50% formamide, 5× SSPE (50 mM NaH_2_PO_4_ pH 7.4, 3750 mM NaCl, 5 mM EDTA, 0.125% SDS), 1× Denhardt’s solution (0.2 g/L Ficoll 400, 0.2 g/L polyvinylpyrrolidone, 0.2 g/L BSA). Biotinylated DNA probe (for *MET16* mRNA: 5′-Biotin-GGACGTTCGAGCAGGTTAAACAGTATATAGATGCAAACAA-3′) were added at 10 nM and hybridized at 65 °C for 1 h followed by overnight incubation at 37 °C. Membranes were washed twice with 2× SSC and 0.1% SDS at room temperature and twice with 0.2× SSC and 0.1% SDS at 37 °C for 20 min each. Detection was performed using the Chemiluminescent Nucleic Acid Detection Module (Thermo Fisher, 89880) following the manufacturer’s protocol.

### Ribosome profiling.

300 mL of yeast culture were grown in YPL to OD 600 ~ 0.8 and harvested, or switched to SL for 1 h and harvested. At time of harvesting, cells were poured into a pre-warmed filtration apparatus and filter through a 0.45 μM cellulose nitrate membrane and immediately scraped into a 50 mL conical tube containing liquid nitrogen. Frozen cells were combined with polysome lysis buffer without cycloheximide, dripped into the liquid nitrogen, and then subjected to cryogrinding, as described^43^. The resulting pulverized cells were thawed on ice and clarified by centrifugation at 20,000×g for 30 min at 4°C. The supernatant was transferred to new tubes and frozen in liquid nitrogen before long-term storage at −80°C. Ribosome profiling was performed as described^44^, with modifications. Briefly, total RNA was quantified with the Quant-iT RiboGreen RNA kit (R11490, ThermoFisher) relative to the rRNA reference standard provided with the kit and fluorescence was measured by GloMax-Multi Jr Detection System (E6070, Promega). Lysates containing 30 μg of RNA were digested with P1 nuclease (30 U/μg RNA) for 1 h at 30°C with shaking at 250 rpm. Ribosome protected fragments were pelleted via sucrose cushion in an Optima TLX Ultracentrifuge with a TLA 110 rotor at 100,000 RPM for 1 h at 4°C. Pelleted RNA was size selected via mirRICH enrichment, as described^44,45^.

For cDNA synthesis and library preparation, 100–500 ng of mirRICH-purified RNA was used as the input for cDNA synthesis, using the improved ordered two-template relay protocol (OTTR v2^46^). Modifications to OTTR v1 include dilution of input RNA to 9 μL, followed by the sequential addition of 2 μL buffer 1A, 1 μL buffer 1B2, and 10μM TurboTail Pol (BoMoC W403AF753A). The reaction was incubated for 2 h at 30°C. Next, 1 μL of buffer 2 and 0.5 μL of rSAP (New England Biolabs, M0371S) premixed with 0.5 μL of 50% glycerol were added. After a 15 min incubation at 37°C, 1 μL of buffer 3 was added, followed by 5 min at 65°C, after which the reaction was placed on ice. Subsequently, 1 μL of buffer 4A, 1 μL of buffer 4B2, and 1 μL of 10 μM Relay Pol were added, followed by incubation for 30 min at 37°C. Reverse transcriptase activity was inactivated by a 5 min incubation at 70°C.

After cDNA synthesis, protein and RNA were removed by RNase A, RNase H, and proteinase K digestion and cDNA products were recovered by Zymo Oligo Clean & Concentrator (Zymo Research, D4061). cDNA was size selected on an 8% denaturing polyacrylamide gel, with imaging to detect IR800 as described^44^. cDNA was quantified using qPCR^43^ and libraries were constructed with 13 amplification cycles using Illumina dual indexing primers (UCSF CAT) and Q5 polymerase. PCR products were purified by Zymo DNA Clean and Concentrator columns followed by AMPure XP (Beckman, A63880) size selection. DNA libraries were pooled according to concentrations provided by TapeStation (Aligent). Libraries were sequenced on an Illumina NovaSeq X with single-end 100 bp reads (UCSF CAT). Sequencing data were processed using cutadapt to trim adapter sequences (constant AGATCGGAAGAGCACACGTCTGAACTCCAGTCAC and 7mer UMI) and deconvolve multiplexed libraries (doi://10.14806/ej.17.1.200). Trimmed reads were aligned by bowtie2 to remove rRNA reads followed by splice-aware genomic alignment of non-rRNA by hisat2. Translation efficiencies were determined with DESeq2.

### Proteomics.

4D-DIA proteomics analysis was performed by Creative Proteomics. Yeast cells were lysed in BPP buffer (100 mM EDTA, 50 mM borax, 50 mM vitamin C, 30% sucrose, 100 mM Tris-HCl, 1% Triton X-100, 1–5% PVPP, and 5 mM DTT, pH 8.0) supplemented with protease inhibitors. Cells were disrupted at −40 °C (4X120 s cycles), and proteins were extracted with Tris-saturated phenol (pH 8.0). After centrifugation, the phenolic phase was re-extracted with BPP buffer, and proteins were precipitated overnight with 0.1 M ammonium acetate in methanol at −20 °C. Pellets were washed with 90% acetone, air-dried, and dissolved in 8 M urea. Protein concentrations were determined using the BCA assay. Protein digestion was performed using the iST Sample Preparation Kit (PreOmics) following the manufacturer’s instructions. Briefly, samples were heated at 95 °C for 10 min, digested with trypsin at 37 °C for 2 h, and peptides were desalted and dried before LC–MS/MS analysis.

Peptides (200 ng) were analyzed on a timsTOF Pro2 mass spectrometer (Bruker Daltonik) coupled to a nanoElute 2 LC system using a C18 column (8 cm × 75 μm, 1.7 μm, IonOpticks). Separation was performed at 50 °C with a 60 min gradient (2.2–90% solvent B: 80% acetonitrile, 0.1% formic acid) at 300 nL/min. Data were acquired in diaPASEF mode (m/z 400–1,000, 25 × 25 Th windows, collision energy 59–20 eV). Raw DIA data were processed in Spectronaut v20.1 (Biognosys) against the *S. cerevisiae* UniProt database using default settings. Trypsin specificity, carbamidomethylation (C, fixed), oxidation (M), and N-terminal acetylation (variable) were applied. A 1% FDR was enforced at all levels, and quantification used the MaxLFQ algorithm. Proteins with fold-change > 1.5 (*p* < 0.05) were considered upregulated and < 1/1.5 (*p* < 0.05) downregulated. 3545 proteins were identified across all samples.

### Cell culture and methionine starvation.

HEK293T and Neuro-2A cells were maintained in a Heracell humidified incubator at 37°C with 5% CO_2_. Both cell lines were cultured in high glucose DMEM (Sigma, D5796) supplemented with 10% fetal bovine serum (Sigma, F6178) and 1X Penicillin-Streptomycin (Gibco, 15140–122). Upon reaching ~80% confluency, cells were washed three times with PBS and then incubated in DMEM (Gibco, 21013–024) containing 10% dialyzed FBS (Gibco, 26400–044), 1% Penicillin-Streptomycin (Gibco, 15140–122), GlutaMAX Supplement (Gibco, 35050–061; final concentration 2 mM), L-cysteine (Sigma, C7352; final concentration 0.2 mM), and with or without L-methionine (Sigma, M9625; final concentration 0.2 mM). After 18 hours, cells were harvested, and total RNA was extracted using TRIzol reagent according to the manufacturer’s protocol.

### Fluorescence *in situ* hybridization (FISH).

RNA FISH was performed using Stellaris RNA FISH probes with CAL Fluor Red 610 Dye (LGC Biosearch Technologies, set of 29 probes) according to the manufacturer’s protocol with minor modifications. Cells were cultured in appropriate media to OD600 of 0.4. Fixation was performed by adding 5 mL of 37% formaldehyde (Electron microscopy sciences, 15686) directly to 45 mL of culture and incubating for 45 min at room temperature. Cells were pelleted at 1600×g for 4 min, washed once with ice-cold fixation buffer (1.2 M sorbitol, 0.1 M K_2_HPO_4_, pH 7.5), and resuspended in 1 mL fixation buffer containing 10 μl Zymolyase 100T. Digestion was proceeded at 30°C for around 45 min until >80% of cells appear phase dark. Digested cells were pelleted at 400 ×g for 5 min and washed twice with ice-cold fixation buffer. Permeabilization was performed by resuspension in 70% ethanol and storage at 4 °C overnight. Prior to hybridization, cells were pelleted and resuspended in hybridization buffer (10% formamide in Stellaris hybridization buffer) containing 125 nM Stellaris probe set targeting the RNA of interest (*MET16*). Hybridization was carried out at 30°C overnight in the dark. Following hybridization, cells were washed with wash buffer A (10% formamide in 1X wash buffer A) for 30 min at 30°C, stained with 5 ng/mL DAPI (ThermoFisher, 62248) in wash buffer A for 30 min at 30°C, and rinsed briefly in wash buffer B. Cells were resuspended in Vectashield mounting medium and imaged immediately.

### *In vitro* transcription of capped RNAs.

DNA template containing a T7 promoter was amplified by PCR. *In vitro* transcription was performed using T7 RNA polymerase (Promega, P2075) in a 50 μl reaction containing 1X Transcription Optimized Buffer (Promega, P2075), 10 mM DTT, 50 U rRNasin (Promega, N2511), 0.5 mM each of rATP, rUTP, and rCTP, 0.05 mM rGTP, 0.5 mM cap analog (m^7^GpppG or GpppG), 5 μg DNA template, and 40 U T7 RNA polymerase. The reaction was incubated at 37 °C for 1 h, after which an additional 40 U of T7 RNA polymerase was added and incubation continued for another 1 h. The reaction was then treated with DNase I and purified using the RNA Clean & Concentrator-25 kit (Zymo Research, R1017).

### Statistical analysis.

Statistical analyses for Fig. 1I and 3H were conducted in Excel using the two-tailed, unpaired Student’s t-test assuming equal variance. Enrichment analysis was conducted using YeastEnrichr^47^. Overrepresentation of gene sets was assessed by Fisher’s exact test, and p values were adjusted for multiple hypothesis testing using the Benjamini–Hochberg false discovery rate procedure.

### *In vitro* translation with yeast extract.

Cell-free translation was performed using yeast extracts prepared as previously described^48^. Briefly, yeast cells were grown in YPD to mid-log phase (OD_600_ = 0.6–0.8) and harvested by centrifugation. Cell pellets were resuspended in buffer A (30 mM HEPES-KOH pH 7.4, 100 mM KOAc, 3 mM Mg(OAc)_2_, and 1 mM DTT) with 85 g/L mannitol at 2:3 volume (ml):pellet weight (g) ratio and lysed using a cryomill. Lysates were centrifugated at 30,000 ×g for 15 min at 4 °C. The middle fraction was transferred into a new tube and centrifuged again at 100,000 ×g for 35 min at 4°C. The middle fraction was collected and desalted into buffer A with 20% glycerol using PD-10 columns (Cytiva, 17085101) while monitoring RNA concentrations. RNA-rich fractions were collected and flash-frozen in liquid nitrogen.

*In vitro* translation reactions were assembled in 15 μL volume with 7.5 μL of yeast translation extract, 250 ng of *in vitro* transcribed RNA, 50 mM KOAc, 2 mM Mg(OAc)_2_, 10 μM of amino acid mix, 0.4 U/μL RiboLock (ThermoFisher, EO0381), 0.06 U/μL creatine kinase (BioVision, P1301), and 1X energy mix containing 20 mM HEPES (pH 7.6), 1 mM ATP, 0.1 mM GTP, 20 mM creatine phosphate, and 2 mM DTT. Reactions were incubated at room temperature for 2 h and stopped by adding SDS loading dye. Translation efficiency was assessed by Western blot.

## Supplementary Material

Supplement 1 **Data S1. (separate file)** Percent methylation from m^7^G-IP, percent input from Cap-Tag, and high confidence targets

Supplement 2 **Data S2. (separate file)** ChIP-seq results

Supplement 3 **Data S3. (separate file)** Ribo-seq results

Supplement 4

## Figures and Tables

**Key resources tables:** 

Yeast strains			
Name		Genotype	References
*wild type*		*MATa CEN.PK*	^37^
*TIR1*		*MATa ho:: TIR1::KanMX6*	This study
ABD1-AID*		MATa ho:: TIR1::KanMX6 ABD1-AID*-1XHA::HYG	This study
ABD1-AID*cho2Δ		MATa ho:: TIR1::KanMX6 ABD1-AID*-1XHA::HYG cho2:: NAT	This study
*pTEF1-ABD1-HA*		*MATa NAT-P_TEF1_ ABD1-6XHA::kanMX6*	This study
*dxo1Δ*		*MATa dxo1:: kanMX6*	This study
*rai1Δ*		*MATa rai1::HYG*	This study
*rai1Δdxo1Δ*		*MATa rai1:: HYG dxo1::kanMX6*	This study
*pTEF1-RAI1*		*MATa HYG-P_TEF1_ RAI1-6XHA::kanMX6*	This study
*pTEF1-DXO1*		*MATa HYG-P_TEF1_ DXO1-6XHA::KanMX6*	This study
*pTEF-RAI1 pTEF-DXO1*		*MATa HYG-P_TEF1_ DXO1-6XHA:: KanMX6 NAT-P_TEF1_ RAI1-6XHA: ble*	This study
DCP2-AID*		MATa ho:: TIR1::KanMX6 DCP2-AID*-1XHA::HYG	This study
*dcs1Δ*		*MATa dcs1:: kanMX6*	This study
*dcs2Δ*		*MATa dcs2:: kanMX6*	This study
*MET16-MS2 Nic96-mCherry*		*MATa ho:: MS2CP::KanMX6 MET16-MS2 NIC96-mCherry:: NAT*	This study
*MET16-MS2 Dhh1-mCherry*		*MATa ho:: MS2CP::KanMX6 MET16-MS2 DHH1-mCherry:: NAT*	This study
*jlp1Δ*		*MATa jlp1:: HYG*	This study
*rpn1Δ*		*MATa rpn1:: HYG*	This study
*gis1Δ*		*MATa gis1:: HYG*	This study
*tpa1Δ*		*MATa tpa1:: HYG*	This study
*jhd2Δ*		*MATa jhd2:: HYG*	This study
*jhd1Δ*		*MATa jhd1:: HYG*	This study
*ecm5Δ*		*MATa ecm5:: HYG*	This study
*PAB1-GFP*		*MATa PAB1-GFP:: KanMX6*	This study
*PUB1-GFP*		*MATa PUB1-GFP:: KanMX6*	This study
*DED1-GFP*		*MATa DED1-GFP:: KanMX6*	This study
*DCP2-GFP*		*MATa DCP2-GFP:: KanMX6*	This study
*DHH1-GFP*		*MATa DHH1-GFP:: KanMX6*	This study
*EDC3-GFP*		*MATa EDC3-GFP:: KanMX6*	This study
*BET1-MS2 Dhh1-mCherry*		*MATa ho:: MS2CP::KanMX6 BET1-MS2 DHH1-mCherry:: NAT*	This study
*RPN12-MS2 Dhh1-mCherry*		*MATa ho:: MS2CP::KanMX6 RPN12-MS2 DHH1-mCherry:: NAT*	This study
*ABD1-HA*		*MATa ABD1-6XHA:: KanMX6*	This study
*HO-GEV-NSP14-HA*		*MATa ho::GEV-NSP14-6xHA::NAT*	This study
*HO-GEV-NSP14-GFP*		*MATa ho::GEV-NSP14-GFP::NAT*	This study
*set2Δ*		*MATa set2:: KanMX6*	^25^
*H3K36A*		*HHT1-HHF1-H3K36A::hygMX, HHT2-HHF2-H3K36A::natMX*	^25^
*HOG1-HA*		*MATa HOG1-6XHA:: KanMX6*	This study
*SLT2-HA*		*MATa SLT2-6XHA:: KanMX6*	This study
HOG1-AID*		MATa ho:: TIR1::KanMX6 HOG1-AID*-1XHA::HYG	This study
SLT2-AID*		MATa ho:: TIR1::KanMX6 SLT2-AID*-1XHA::HYG	This study
*HOG1-FLAG*		*MATa HOG1-3XFLAG:: KanMX6*	This study
*SLT2-FLAG*		*MATa SLT2-3XFLAG:: KanMX6*	This study
Plasmids			
Name	Description	References
pFA6a-Hyg		To knock out a gene of interest (GOI) with the Hyg gene	^38^
pFA6a-KanMX6		To knock out a GOI with the KanMX6 gene	^38^
pFA6a-HA-KanMX6		To tag a GOI with 6XHA tag, kanamycin resistance	This study
pFA6a-HA-Hyg		To tag a GOI with 6XHA tag, hygromycin resistance	This study
pFA6a-HA-ble		To tag a GOI with 6XHA tag, phleomycin resistance	This study
pFA6a-FLAG-Nat		To tag a GOI with 3XFLAG tag, natamycin resistance	This study
pFA6a-TEF1-OM-KanMX6		To insert the TEF1 promoter in front of the CDS of a GOI, kanamycin resistance	This study
pFA6a-TEF1-OM-Nat		To insert the TEF1 promoter in front of the CDS of a GOI, natamycin resistance	This study
HO-NSP14–6XHA-Nat-HO		To insert NSP14–6XHA at the HO locus	This study
HO-NSP14-yeGFP-Nat-HO		To insert NSP14-yeGFP at the HO locus	This study
pFA6a-yeGFP-KanMX6		To tag a GOI with yeGFP tag	This study
pFA6a-NES-yeGFP-KanMX6		To tag a GOI with NES-yeGFP tag	This study
pNHK53		Template for osTIR1 PCR	^24^
pHyg-AID*		Template for AID* PCR	^24^
HO-OsTIR1-KanMX6-HO		To insert OsTIR1 at the HO locus	This study
pHyg-AID*-HA		To tag a gene of interest with AID*-1XHA tag	This study
pET296-YcpLac111 CYC1p-MCP-NLS-2xyeGFP		Template for CYC1P-MCP-NLS-2xyeGFP PCR	^31^
HO-CYC1p-MCP-NLS-yeGFP-Hyg-HO		To insert CYC1p-MCP-NLS-yeGFP at the HO locus	This study
pET259-pUC57 24xMS2V6		To tag a gene of interest with 24XMS2V6	^31^
pSH65		To induce Cre recombinase expression under GAL promoter	^31^
pACT-GEV		To PCR the ACT-GEV fragment for integration	^39^
FISH probes			
No.	Sequences	Target site	GC content
1	tccaatgatccaactgttct	42	40%
2	tttccagcttgattagttgt	66	35%
3	ccatgcaataatctcctgtg	89	45%
4	aggtgaggaaacgttacgat	112	45%
5	accaaatgcagtggtttgga	134	45%
6	tcgatagtaaccaagccagt	157	45%
7	ttcagatagctttgacaaca	179	35%
8	atagttctggcatgtagtat	201	35%
9	agtttgtgggaaatggtgca	236	45%
10	tctcaatctcgttttttagt	261	30%
11	ggtttgatttttaggctggt	290	40%
12	catccggcttatatacgtga	312	45%
13	aaaatctgcctccgattcac	335	45%
14	agaaatccccgtatttcgag	357	45%
15	acttgtcatcatctttctcc	381	40%
16	tccactttggccagataatc	403	45%
17	tttgtaggcacgatgtgcag	425	50%
18	acacagcacttatatgtagc	447	40%
19	tgtgattttcttctaccagt	469	35%
20	ataatcgacagttgggagcg	499	50%
21	agattccattaagttcgtct	525	35%
22	cgtccaattgatcaatggat	554	40%
23	tatactgtttaacctgctcg	576	40%
24	tgtatggtacattgtttgca	600	35%
25	gatctatatccaaggtccaa	628	40%
26	gtgtggaatggtaatcacca	651	45%
27	tcatcttcaccttccttgac	676	45%
28	ttcatgaattccacactcgg	728	45%
29	aaaattgcgcgaatcggctg	750	50%

## Data Availability

All data necessary for evaluating the conclusions are presented in the article and its supplemental materials. Primary data will be deposited at a publicly accessible repository. Sequencing data have been deposited at GEO with accession numbers GSE312836, 313216, 313217, 313220.
