# Supplementary material for: Metabolic stress reveals widespread accumulation of cap-unmethylated RNAs
Source: bioRxiv. 2026 Feb 24:2026.02.23.707474. Preprint. [Version 1] doi: 10.64898/2026.02.23.707474 (PMC13004159; doi:10.64898/2026.02.23.707474)
Supplement: Supplement 4 [file NIHPP2026.02.23.707474v1-supplement-4.pdf]

bioRxiv preprint doi: https://doi.org/10.64898/2026.02.23.707474; this version posted February 24, 2026. The copyright holder for this preprint (which was not certified by peer review) is the author/funder, who has granted bioRxiv a license to display the preprint in perpetuity. It is made available under aCC-BY 4.0 International license.

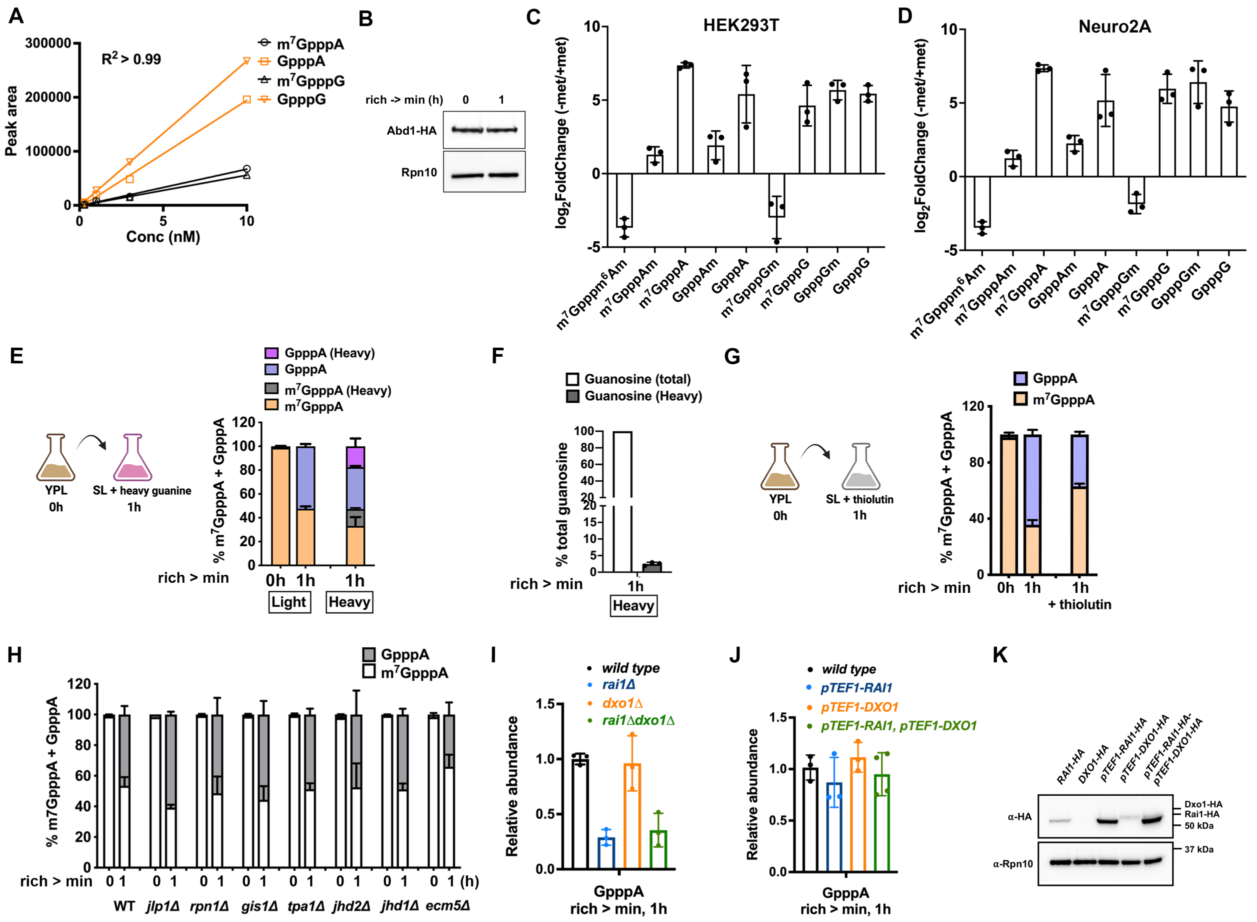

Fig. S1. (A) Representative standard curves generated using purified cap analogs. **(B)** Western blot analysis of Abd1 levels in yeast cells following a switch from rich medium to minimal medium. **(C, D)** LC-MS/MS analysis of the relative abundance of cap structures normalized to guanosine in HEK293T (C) and Neuro2A (D) cells. Fold change was calculated by comparing samples before and after 18 h of methionine starvation. Data represent mean ± SD, n = 3. **(E)** LC-MS/MS analysis of cap dinucleotide percentages with the addition of heavy guanine during a 1 h switch from rich to minimal media. Data represent mean ± SD. **(F)** Percentage of heavy labeled guanosine as compared to light guanosine when cells were grown in minimal medium containing heavy guanine for 1 h. Data are shown as mean ± SD. **(G)** LC-MS/MS analysis of cap dinucleotide percentage with the addition of thiolutin (20 µg/ml) when switching cells from rich to minimal medium for 1 h. Data represents mean ± SD. **(H)** LC-MS/MS analysis of cap dinucleotides percentages in wild type and demethylase deletion strains. Data represents mean ± SD, n=3. **(I, J)** Relative abundance of GpppA cap structures of cells grown in minimal medium for 1 hour. Data were measured by LC-MS/MS and shown as mean ± SD. **(K)** Western blot analysis of strains from (J).

bioRxiv preprint doi: https://doi.org/10.64898/2026.02.23.707474; this version posted February 24, 2026. The copyright holder for this preprint (which was not certified by peer review) is the author/funder, who has granted bioRxiv a license to display the preprint in perpetuity. It is made available under aCC-BY 4.0 International license.

bioRxiv preprint doi: https://doi.org/10.64898/2026.02.23.707474; this version posted February 24, 2026. The copyright holder for this preprint (which was not certified by peer review) is the author/funder, who has granted bioRxiv a license to display the preprint in perpetuity. It is made available under aCC-BY 4.0 International license.

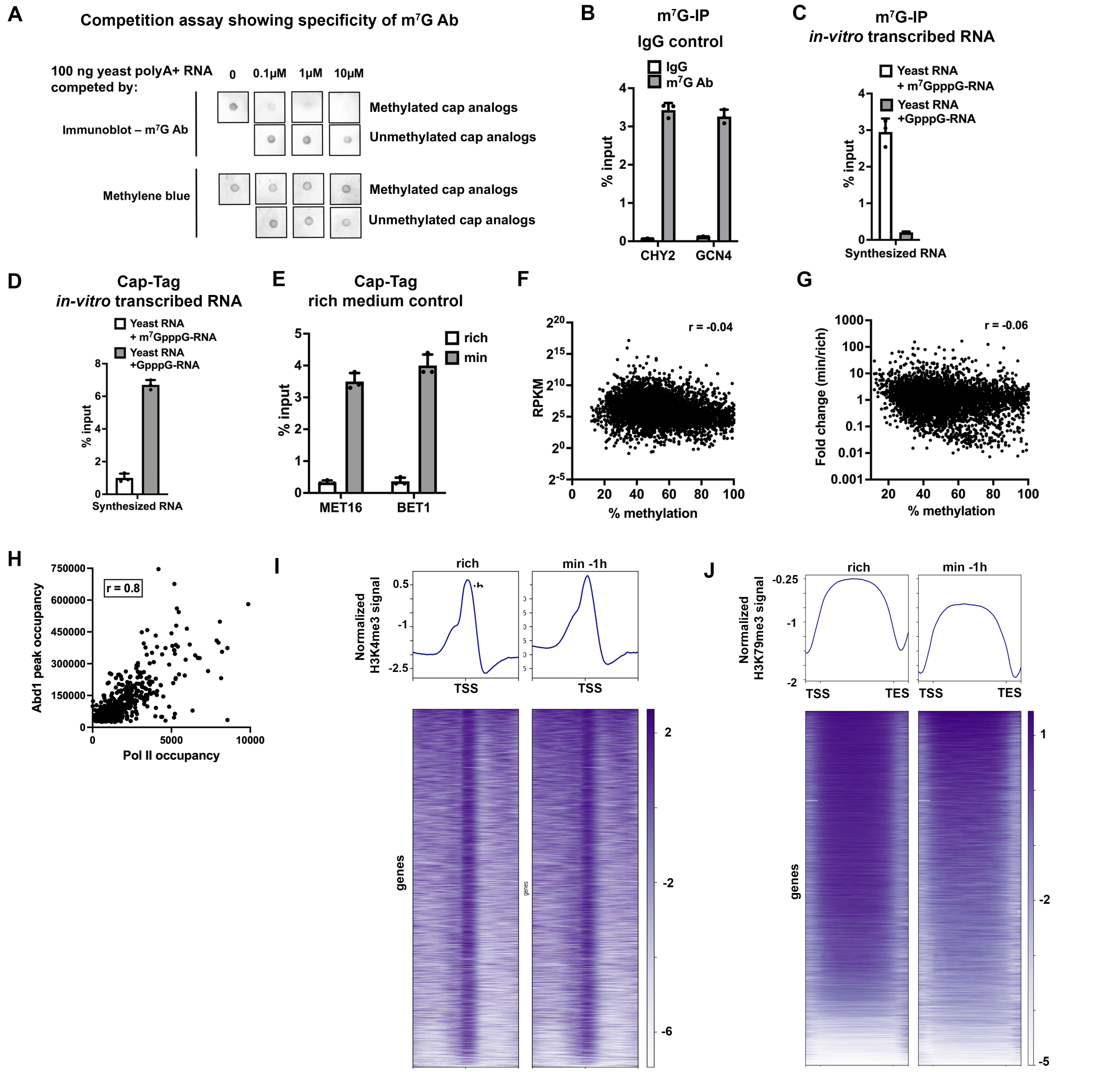

Fig. S2. **(A)** Dot blot analysis of m^{7}G cap in yeast mRNAs extracted from cells grown in rich medium, using either a mixture of methylated cap analogs (m^{7}GpppA and m^{7}GpppG) or unmethylated cap analogs (GpppA and GpppG) as competitors for antibody binding. **(B)** Comparison of m^{7}G-IP-qPCR results for mRNAs extracted from cells grown in rich medium, using IgG as a control. Percent input was measured for two randomly selected mRNAs, CHY2 and GCN4. Data represents mean ± SD, n=3. **(C)** m^{7}G-IP-qPCR results of *in vitro*–transcribed and capped RNAs mixed with 100 ng of yeast mRNAs. Data represents mean ± SD, n=3. **(D)** Cap-Tag-qPCR results of *in vitro*–transcribed and capped RNAs mixed with 100 ng of yeast mRNAs. Data represents mean ± SD, n=3. **(E)** Cap-Tag-qPCR results of mRNAs extracted from cells grown in rich or minimal media. Percent input was measured for two lowly-methylated mRNAs identified in the m^{7}G-IP experiment. **(F, G)** Correlation of m^{7}G methylation levels (measured by m^{7}G-IP) with (F) Reads Per Kilobase of transcript per Million mapped reads (RPKM), (G) fold change in mRNA levels between minimal and rich media. **(H)** Correlation between Abd1 and Rpb1 (Pol II) occupancy. **(I, J)** ChIP-seq profiles displaying H3K4me3 (I) and H3K79me3 (J) distribution in cells grown in rich or minimal medium for 1 h, normalized to total H3 ChIP signals.

bioRxiv preprint doi: https://doi.org/10.64898/2026.02.23.707474; this version posted February 24, 2026. The copyright holder for this preprint (which was not certified by peer review) is the author/funder, who has granted bioRxiv a license to display the preprint in perpetuity. It is made available under aCC-BY 4.0 International license.

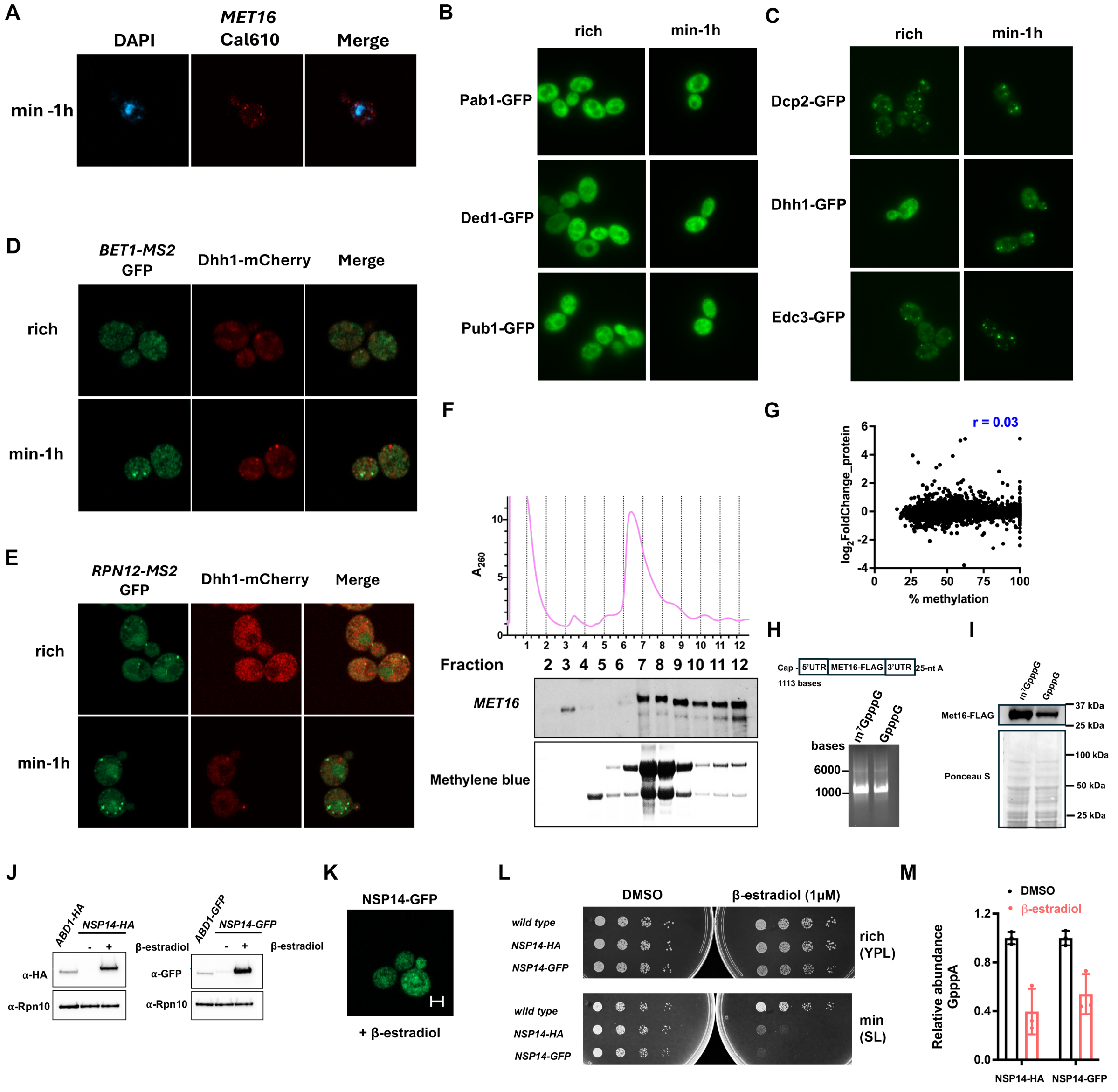

Fig. S3. (A) Microscopy images of yeast cells after a 1 h shift to minimal medium. Blue indicates DAPI staining of DNA; red indicates RNA FISH signal for *MET16* detected with Cal Fluor Red 610 dye. **(B)** Microscopy images of GFP-tagged yeast stress granule markers—Pab1, Ded1, and Pub1—before and after a 1 h shift to minimal medium. **(C)** Microscope images of GFP-tagged yeast P-body markers including Dcp2, Dhh1, and Edc3, before and after 1 h switch for minimal medium. **(D, E)** Microscopy images of yeast cells before and after a 1 h shift to minimal medium. Green indicates MCP-GFP bound to *BET1-MS2* (d, % methylation = 33) or *RPN12-MS2* (e, % methylation = 29) mRNAs; red indicates the P-body marker Dhh1-mCherry. **(F)** Northern blot analysis of *MET16* mRNA using RNA extracted from polysome fractions. Polysome profiling was performed on cells grown in minimal medium for 1 h. Equal proportions of each fraction were loaded, and RNA concentrations were not normalized. **(G)** Correlation of $\log_2$FoldChange of protein levels (min 1h/rich) and % methylation levels of each mRNA in cells grown in minimal medium for 1 h. Pearson r = 0.03. **(H)** RNA gel electrophoresis of *in vitro* transcribed *MET16* RNAs with either m^7pppG or GpppG cap. **(I)** Western blot analysis of *in vitro* translated of *MET16* RNAs using cell-free yeast translation extract. Ponceau S stain confirms equal loading of yeast

bioRxiv preprint doi: https://doi.org/10.64898/2026.02.23.707474; this version posted February 24, 2026. The copyright holder for this preprint (which was not certified by peer review) is the author/funder, who has granted bioRxiv a license to display the preprint in perpetuity. It is made available under aCC-BY 4.0 International license.

extracts. **(J)** Western blot showing overexpression of Nsp14-HA and Nsp14-GFP following induction with 1 μM β-estradiol for 1 h in minimal medium. Rpn10 was used as a loading control. **(K)** Microscopy image of yeast cells expressing NSP14-GFP. Scale bar indicates 2 μm. **(L)** Serial dilution assays of wild-type, *NSP14-HA*, and *NSP14-GFP* strains spotted on rich or minimal media supplemented with DMSO or 1 μM β-estradiol. **(M)** LC-MS/MS analysis of relative GpppA mRNA levels in *NSP14-HA* and *NSP14-GFP* cells with or without 1 μM β-estradiol.

bioRxiv preprint doi: https://doi.org/10.64898/2026.02.23.707474; this version posted February 24, 2026. The copyright holder for this preprint (which was not certified by peer review) is the author/funder, who has granted bioRxiv a license to display the preprint in perpetuity. It is made available under aCC-BY 4.0 International license.

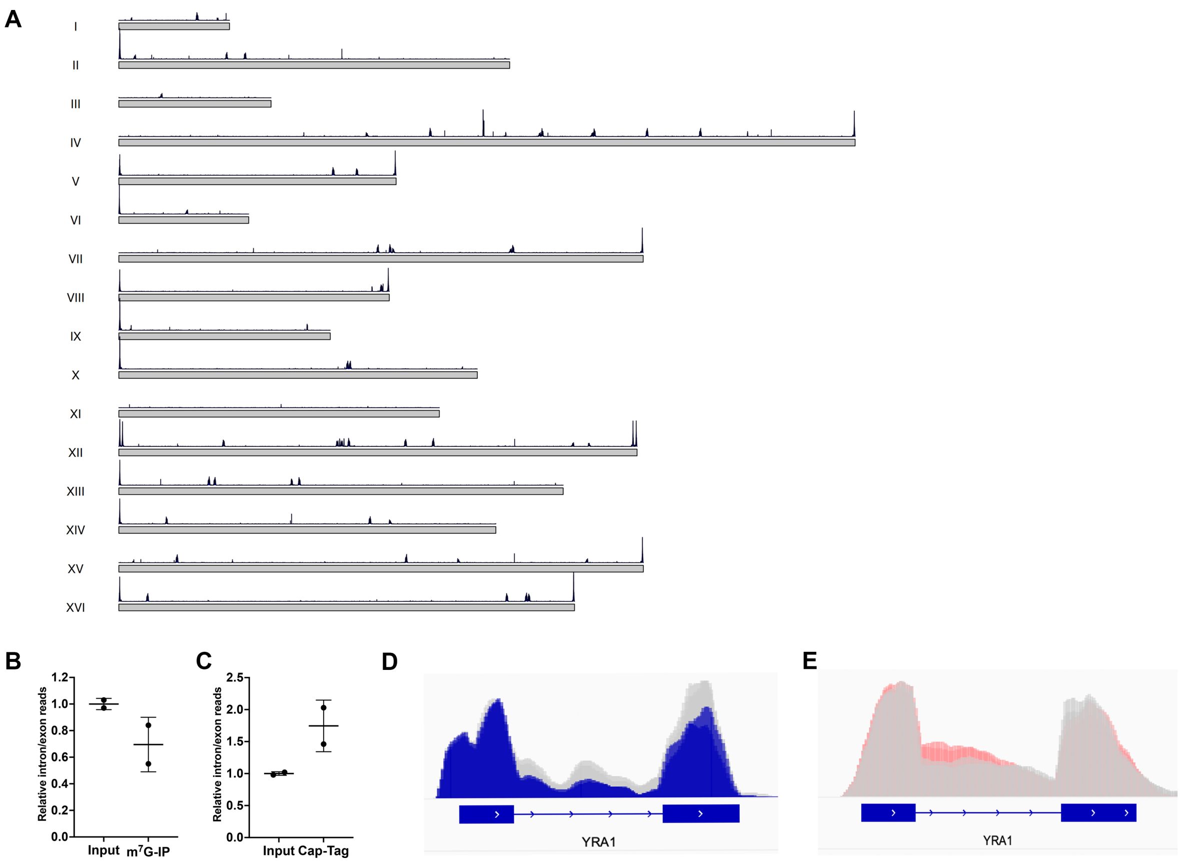

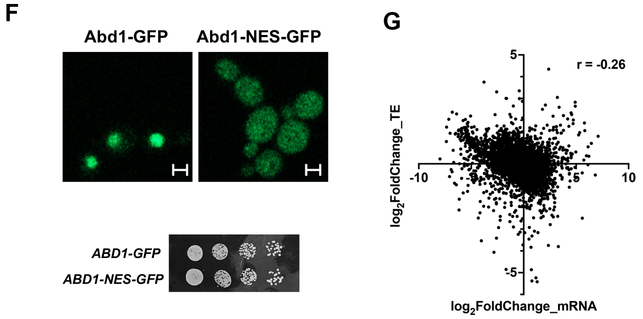

H

Gene	Log_2fc_TE	Log_2fc_mRNA	% me	Function
JLP1	3.1	3.2	26	Sulfur metabolism
GTO1	2.3	4.6	37	Oxidative stress_glutathione
YKL065W-A	2.2	2.8	36	Unknown
RMD6	2.1	4.2	31	Sporulation
CUP1-1	1.8	4.7	35	Oxidative stress_copper
ISA1	1.7	0.6	40	Mitochondria_Fe-S cluster
ARG3	1.6	1.9	28	Arginine biosynthesis
MET16	1.5	5.8	26	Sulfur metabolism
COX26	1.5	0.6	32	Mitochondria_respiration
MUP3	1.4	5.3	37	Sulfur metabolism
GRX8	1.4	3.2	37	Oxidative stress_glutathione
SHH3	1.4	3.6	21	Mitochondria_unknown
CUP1-2	1.3	5.2	34	Oxidative stress_copper
PIC2	1.2	1.2	35	Mitochondria_transport
YIR042C	1.2	1.1	39	Unknown

Fig. S4. (A) Chromosome coverage plot showing distribution of H3K36me3 peaks across chromosomes in cells grown in minimal medium for 1h. **(B)** Ratio of intronic to exonic reads in input and m^{7}G-IP samples; n=2. **(C)** Ratio of intronic to exonic reads in input and Cap-Tag samples; n=2. **(D, E)** RNA-seq coverage tracks of *YRA1* visualized in Integrative Genomics Viewer (IGV). Gray, input; blue, m^{7}G-IP; pink, Cap-Tag. Coverage tracks were generated using bamCoverage (deepTools) and normalized to CPM(counts per million). **(F)** Microscopy images of yeast strains expressing Abd1-GFP and ABD1-NES-GFP grown in rich medium. Scale bar shows 2 µM. Growth assay of ABD1-GFP and ABD1-NES-GFP strains on YPL (rich) plates. **(G)** Correlation between log_2 fold change in TE vs. log_2 fold change in mRNA levels. Pearson r = -0.26. **(H)** mRNAs with low cap methylation (% methylation < 40), increased expression (log_2 fold change > 0), and enhanced translation efficiency (log_2 fold change in TE > 1) after switching from

bioRxiv preprint doi: https://doi.org/10.64898/2026.02.23.707474; this version posted February 24, 2026. The copyright holder for this preprint (which was not certified by peer review) is the author/funder, who has granted bioRxiv a license to display the preprint in perpetuity. It is made available under aCC-BY 4.0 International license.

rich to minimal medium. Orange, pink, and blue highlight genes involved in sulfur metabolism, oxidative stress response, and mitochondrial function, respectively.

bioRxiv preprint doi: https://doi.org/10.64898/2026.02.23.707474; this version posted February 24, 2026. The copyright holder for this preprint (which was not certified by peer review) is the author/funder, who has granted bioRxiv a license to display the preprint in perpetuity. It is made available under aCC-BY 4.0 International license.

Supplemental Data (Spreadsheets):

Data S1. (separate file) Percent methylation from m^7G-IP, percent input from Cap-Tag, and high confidence targets

Data S2. (separate file) ChIP-seq results

Data S3. (separate file) Ribo-seq results
